# Effect of Rolling Temperature on Microstructure Evolution and Mechanical Properties of AISI316LN Austenitic Stainless Steel

**DOI:** 10.3390/ma11091557

**Published:** 2018-08-29

**Authors:** Yi Xiong, Yun Yue, Tiantian He, Yan Lu, Fengzhang Ren, Wei Cao

**Affiliations:** 1 School of Materials Science and Engineering, Henan University of Science and Technology, Luoyang 471023, Henan, China; luyan@haust.edu.cn (Y.L.); renfz@haust.edu.cn (F.R.); 2 Collaborative Innovation Center of Nonferrous Metals, Luoyang 471023, Henan, China; 3 National United Engineering Laboratory for Advanced Bearing Tribology, Henan University of Science and Technology, Luoyang 471023, Henan, China; yueyunbw@163.com (Y.Y.); tthe@haust.edu.cn (T.H.); 4 Nano and Molecular Systems Research Unit, University of Oulu, 90014 Oulu, Finland; Wei.Cao@oulu.fi; 5 School of Mechanical and Automotive Engineering, Anhui Polytechnic University, Wuhu 241000, Anhui, China

**Keywords:** 316LN austenitic stainless steel, strain-induced martensitic transformation, deformation mechanism, K–S relationship

## Abstract

The impacts of rolling temperature on phase transformations and mechanical properties were investigated for AISI 316LN austenitic stainless steel subjected to rolling at cryogenic and room temperatures. The microstructure evolution and the mechanical properties were investigated by means of optical, scanning, and transmission electron microscopy, an X-ray diffractometer, microhardness tester, and tensile testing system. Results showed that strain-induced martensitic transformation occurred at both deformation temperatures, and the martensite volume fraction increased with the deformation. Compared with room temperature rolling, cryorolling substantially enhanced the martensite transformation rate. At 50% deformation, it yielded the same fraction as the room temperature counterpart at 90% strain, while at 70%, it totally transformed the austenite to martensite. The strength and hardness of the stainless steel increased remarkably with the deformation, but the corresponding elongation decreased dramatically. Meanwhile, the tensile fracture morphology changed from a typical ductile rupture to a mixture of ductile and quasi-cleavage fracture. The phase transformation and deformation mechanisms differed at two temperatures, with the martensite deformation contributing to the former, and austenite deformation to the latter. Orientations between the transformed martensite and its parent phase followed the K–S (Kurdjumov–Sachs) relationship.

## 1. Introduction

As one of the key stainless steels (SSs), 316LN austenitic SSs (ASS) possess outstanding anticorrosion and antioxidation merits, yet are widely used in various fields such as petroleum, chemical industry, and nuclear power plants [1,2]. However, the rather low yield strength, only 200 MPa after solid solution treatment, demands additional treatments to broaden its application fields. It has been noticed that its mechanical properties can be enhanced by grain refinement, phase transformation strengthening, and work hardening [3,4,5]. As a result, high ductility and high yield strength have been introduced into the treated ASS. For instance, during cold rolling, the stable ASS (e.g., 310S ASS) can maintain the austenitic structure after deformation [6,7]. As for the meta-stable ASS such as 304 and 316 ASS [8,9], martensite transformation (i.e., strain-induced phase transformation) turns up easily during deformation, and leads to a dual-phase microstructure containing austenite and martensite at the same time. The occurrence of the new structure significantly improves the work-hardening ability of ASS and substantially enhances its strength. Studies of martensite transformation during deformation at room temperature have indicated that the volume fraction of transformed martensite increases with the strain, but decreases with the strain rate [10]. Compared with torsion and compression, tensile deformation is more effective at inducing martensite transformation [11]. Powell et al. [12] investigated the effect of temperature (10–293 K), strain rate, and deformation modes such as tension, torsion, and compression on the strain-hardening characteristics of type 301 and 304 ASS. The martensite fractions in each treatment were revealed, and their observations suggest that the deformation mode substantially affects the transformation of martensite in the plastic range, and uniaxial tension enhances martensite formation when compared to the compression or torsion routes. Thus, both the deformation degree and homogeneity of deformation are key factors for martensite transformation.

Another crucial factor for phase transformation is the deformation temperature. For instance, the volume fraction of strain-induced martensite and the yielded mechanical properties are very different after rolling at temperatures of 273 K and 258 K [4]. With a strain of up to 95%, the volume fraction of transformed martensite (65%) at 258 K was higher than that (50%) deformed at 273 K. Consequentially, the mechanical properties (strength and hardness) from the former were obviously better than the latter. Eskandari [13] investigated the room temperature mechanical properties of 316L ASS after being rolled at a strain range of 10% to 95% at the temperature range of 0–77 K. The austenite was completely transformed into martensite at low temperature. Interestingly, smaller strains were needed to accomplish martensite transformation at lower temperatures. In our latest work [14], we also found that cryorolling could promote the transformation of austenite into martensite. After cryorolling, the martensite grains were refined to nanometer scaled particles, and the strength significantly improved. Similar material behaviors were also revealed in the studies of the influence of deformation temperature on the microstructural evolution of 304L ASS in [15,16,17,18].

Despite the aforementioned achievements, phase transformation mechanisms and mechanical properties are seldom studied for 316LN ASS after deformations under different temperatures and strains. The temperature impacts on phase transformation remain elusive. Herein, we systematically investigated the impacts of deformation temperature on the microstructural evolution and property changes of 316LN ASS subjected to cryorolling and rolling at room temperature. It has generally been found that the formation of martensite and property enhancements are easier to reach through low temperature treatment. Based on the microstructural and property determinations, phase transformation mechanisms were also proposed. Besides providing a route to improve the properties of the meta-stable ASS, the work hopes to serve as a starting point to understand phase transformation schemes at different temperatures.

## 2. Experimental Methods

Hot-rolled AISI 316LN ASS sheets with a thickness of 10 mm were used in this study. The measured compositions (in wt %) were 0.01 C, 0.49 Si, 0.87 Mn, 17.09 Cr, 14.04 Ni, 2.56 Mo, 0.14 N, and balance Fe. The as-prepared materials were solution treated at 1050 °C (±5 °C) for 60 min under an argon atmosphere and subsequently water quenched to room temperature. The microstructure of ASS after solution treatment was a single-phase austenite with the grain size of about 80 μm, as given in [14]. Rectangular samples with a size of 100 × 50 × 5 mm^3^ were cut from the solid-solution sheet for the next rolling process. In cryorolling, samples were soaked in liquid nitrogen (−196 °C) for 10 min to guarantee the temperature before rolling. Then, the samples were taken out quickly for multi-pass rolling operation to a total reduction of 30%, 50%, 70%, and 90%, respectively. The samples were cryorolled in the strain rate range of $\dot{\varepsilon }$ = 0.6~1.5 s^−1^ from 10 to 1 mm thickness, i.e., an accumulated strain of ε = 2.32, with a reduction of ~5% per pass. The obtained cryogenic rolled steel sheets were labeled as CRx, where x refers to the percentage of the strain. Meanwhile, the room temperature rolled sheets were also obtained utilizing the same strains and labeled as RTRx.

The dimensional requirement of micro-tensile specimens can be found in [6]. Uniaxial tensile tests were performed on an Instron 5980 mechanical test system, and fracture surface morphology was conducted using a scanning electron microscope (SEM, JSM-5610LV) at an accelerating voltage of 20 kV. Microstructures were examined via optical microscopy (OM, Zeiss AxioVert A1) and transmission electron microscopy (TEM, JEOL-2100, operated at 200 kV). The samples for OM were etched in a solution that consisted of 0.20 g sodium-metabisulfate in 100 mL distilled water and 10 mL hydrochloric acid in 100 mL distilled water. The etching time varied from 2 to 5 min, depending on the fraction of martensite in each specimen, to enable the martensite phase to be adequately revealed as a dark phase. Thin foils for TEM were prepared by twin-jet electropolishing of 3 mm disks punched from the specimens using a solution of 10% perchloric acid in acetic acid as the electrolyte. X-ray diffraction (XRD) analysis was conducted to determine the phase composition of the rolled ASS on a D8 ADVANCE X-ray diffractometer with Cu–Kα as the incident source. The scan angle was selected from 40° to 100° with a step size of 0.02°. The tube voltage and current were 40 kV and 40 mA, respectively. (200), (220), (311) reflections of the austenite phase (γ) and (200), (211), (220) reflections of the martensite phase (α′) were chosen to calculate the volume fraction of strain-induced martensite (Vα′) at different strains. The formula [19] reads: $V_{\alpha \prime }=\frac{1/n\sum_{j=1}^{n}I_{\alpha \prime }^{j}/R_{\alpha \prime }^{j}}{1/n\sum_{j=1}^{n}I_{\alpha \prime }^{j}/R_{\alpha \prime }^{j}+1/n\sum_{j=1}^{n}I_{\gamma }^{j}/R_{\gamma }^{j}}$.

Here, n denotes the number of selected reflections; I is the diffraction intensity factor; and R is the scattering factor of the materials. To obtain reliable Vα′, three different sites were selected randomly and the mean value was considered as the volume fraction of deformation-induced martensite.

Microhardness was measured using an MH-3 Vickers microhardness tester with 200 g normal load, 10 s loading time, and 5 s holding time on the polished region. An average microhardness value was determined based on five indentation measurements.

## 3. Results

### 3.1. Martensite Transformation

**Figure 1:**
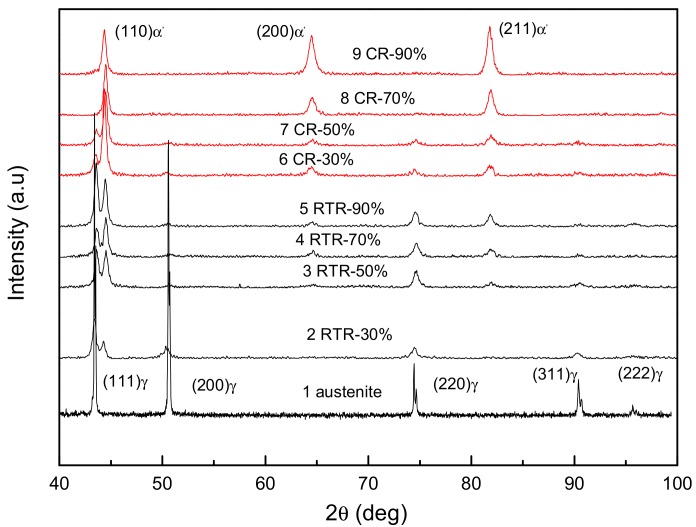
XRD patterns of the 316LN austenitic stainless steel before and after deformation at different temperatures (the data of CR is from [14]).

The XRD patterns of AISI 316LN ASS subjected to room temperature rolling are shown in Figure 1. Compared with these after cryorolling, the diffraction peak intensities of martensite increased with strain under both deformation conditions, while those of austenite decreased. For cryorolling, the XRD signature of austenite disappeared completely at a strain of 70%, leaving martensite the only component [14]. The strain-induced martensite transformation was accomplished. However, the total phase transformation could not be reached through room temperature rolling. Even for RTR90, the diffraction peaks of austenite still coexisted with those of martensite as shown in Figure 1. The room temperature rolled steel had a dual-phase microstructure containing the newly formed martensite and remained austenite.

**Figure 2:**
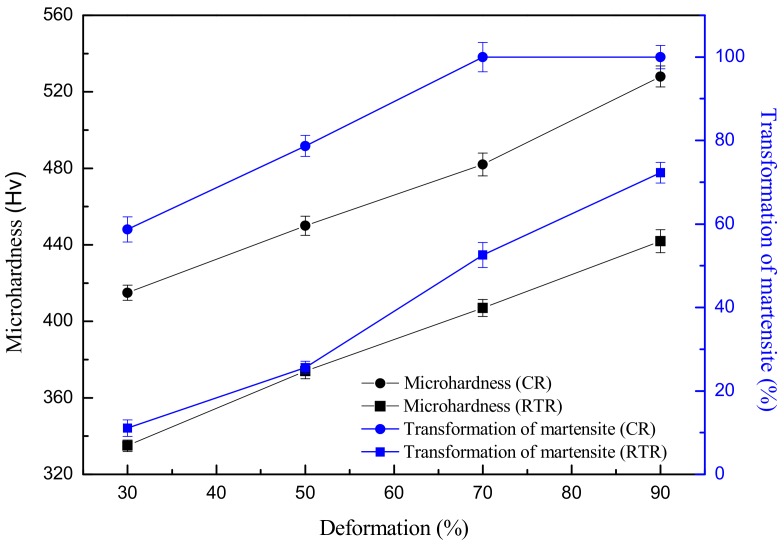
Relationship of the volume fraction of the martensite and microhardness with deformations at different temperatures (the data of CR is from [14]).

Figure 2 shows the volume fraction of martensite and hardness of rolled AISI 316LN ASS as a function of the deformation. Both the volume fraction of martensite and hardness increased with the deformation degree under these deformation conditions. When the deformation was 30%, the volume fraction of transformed martensite was only 11%. An increase in the transformation of martensite followed the deformation degree. When the rolling deformation was up to 90% at room temperature, the volume fraction of transformed martensite increased to 72%. Xu et al. [20] also found the incomplete transformation of strain-induced martensite in AISI 316LN ASS after RTR90, but the volume fraction of transformed martensite was only 46%. The discrepancy between the present work and the work by Xu et al. could mainly be due to the difference in chemical composition. The discrepancy of the chemical composition led to the different stacking fault energy. The stacking fault energy of AISI 316LN ASS in our present paper was lower than that in [20]. The lower the stacking fault energy, the more unstable the austenite, therefore the easier it is for the strain-induced martensite transformation to occur. Thus, at the same deformation, the volume fraction of the transformed martensite in the present paper was higher than that in [20]. Furthermore, the increase of hardness was more significant when compared with the volume fraction of martensite due to the obvious work-hardening of the studied steel. At the same deformation, cryorolling introduced a much higher volume fraction of martensite and hardness than the room temperature rolling. Following Figure 1 and Figure 2, the volume fraction of transformed martensite by cryorolling were calculated as 58.7% and 78.7% under strains of 30% and 50%, respectively [14]. These contents could only be reached at strains up to 70% and 90% under room temperature rolling condition. A similar trend was found in the hardness tests, where the hardness increased from 335 HV to 442 HV when the deformation was 30% and 90%, respectively, denoting that changes of the mechanical property can be mainly attributed to the volume fraction change of strain-induced martensite. It can be seen that during cryorolling, the martensite transformation experienced a surge process consistent with the results reported in Roy et al. [16]. In contrast, the martensite fraction increased gradually during room temperature rolling without an obvious surge process.

### 3.2. Microstructural Evolution

**Figure 3:**
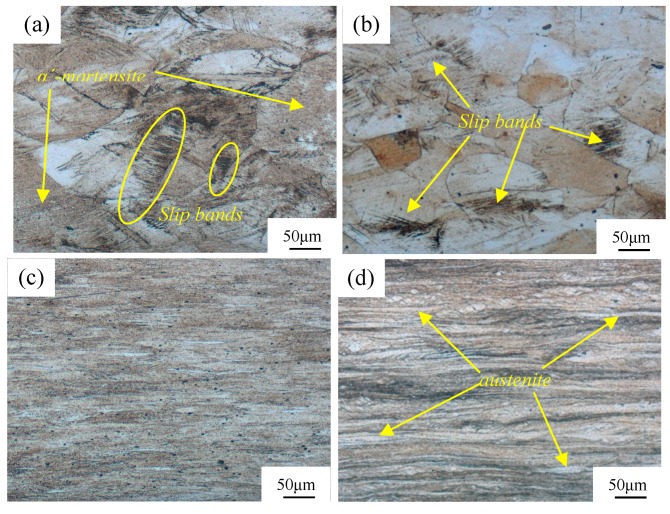
Metallographic structure of the 316LN austenitic stainless steel after CR and RTR: (**a**) CR-30%, (**b**) RTR-30%, (**c**) CR-90%, and (**d**) RTR-90%.

Figure 3 shows the optical micrographs of AISI 316LN ASS subjected to cryorolling and room temperature rolling with different deformation degrees. In CR30, a multiplicity of α′-martensites and slip lines are the main microstructure as shown in Figure 3a. Slip lines are typical signatures of dislocation gliding. In order to accommodate the severe plastic deformation, the austenite grains are deformed in the form of dislocation slips [14]. Due to the smaller deformation of the austenite grains under room temperature rolling condition, the number of slip lines is much less intensive when compared to the former deformation condition. Furthermore, owing to the intensified plastic deformation of α′-martensite, strain-induced martensites are broken up into finer laths, are elongated in the rolling direction, and presented in fiber morphology as shown in Figure 3c. Once rolled in room temperature, the 316LN ASS was associated with the induced martensite and remained austenite, in accordance with the XRD determination shown in Figure 1. The long stripes in Figure 3d clearly show severe plastic deformation of the initial austenite along the rolling direction.

**Figure 4:**
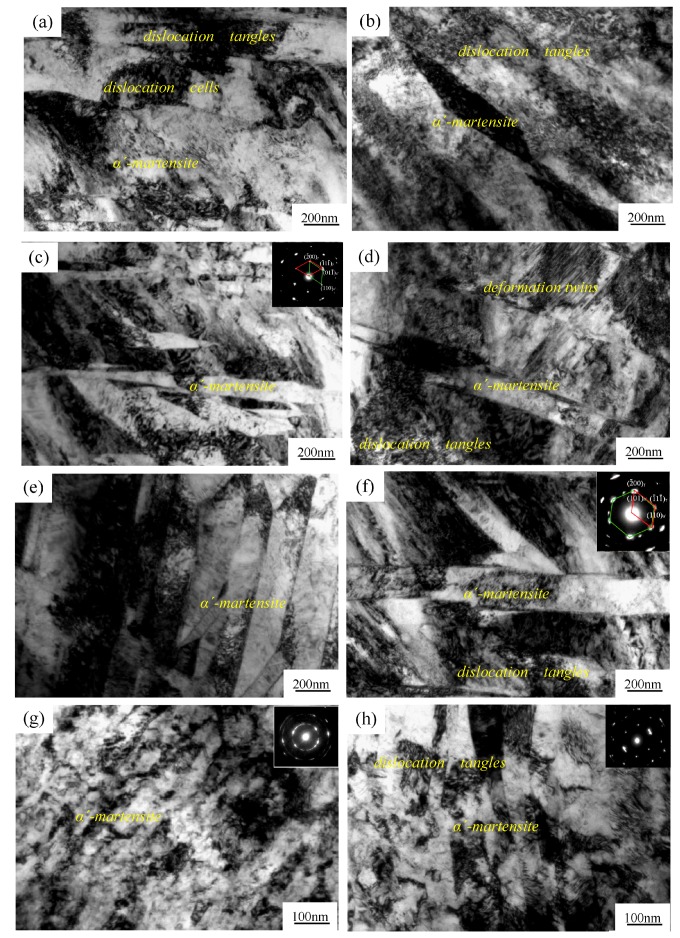
TEM images of the 316LN austenitic stainless steel after CR and RTR: (**a**) CR-30%, (**b**) RTR-30%, (**c**) CR-50%, (**d**) RTR-50%, (**e**) CR-70%, (**f**) RTR-70%, (**g**) CR-90%, and (**h**) RTR-90%.

We further studied the microstructural evolutions of the rolled samples through TEM. As shown in Figure 4a, CR30 was composed of dislocation tangles and even dislocation cells, strain-induced martensite. However, RTR30 mainly consisted of dislocation tangles and just a small amount of strain-induced martensite in local regions. At 50% strain, dislocation tangles and strain-induced martensite were the main features of the cryorolled steel, with the latter content substantially enhanced when compared to the case of CR30. The room temperature rolled sample RTR50 had a similar microstructure to CR30, but also contained some deformation twins. At the strain of 70%, the lath martensite structure (~100 nm width) became the primary content in CR70, while the mixture of lath martensite and dislocation tangles coexisted in RTR70. Of note, the martensite content increased significantly in RTR70. This was due to the additional nucleation sites for martensite transformation as a result of the interactions among the deformation twins, and between the deformation twins and dislocations. At a high strain of 90%, an obvious fragmentation of martensite was observed in the CR90. The selected area electron diffraction (SAED) pattern turned out to be a continuous ring, denoting an even distribution of nanometer-scaled grains. The main microstructure of the RTR90 remained as lath martensite. The lath was refined to a width ranging from 80 nm to 100 nm and the dislocation tangles were retained to some extent at the same time. Similar to Figure 4c, the corresponding diffraction spot was a discontinuous ring, mainly due to the microstructure of both the martensite and austenite.

Several studies [21,22] have shown that metastable austenite consists of many deformation twins or stacking faults before martensitic transformation. During the deformation process, the orientation between the newly generated martensite and parent phase austenite followed the Kurdjumov–Sachs (K–S) orientation relationship. To check the K–S validity in the present study, the microstructures of CR50 and RTR70 were carefully examined. The rolled steels had martensite volume fractions of 78.7% and 52.6%, respectively. By indexing the diffraction patterns in Figure 4c,f, it was found that {111} γ∥{011} α′; $<01\bar{1}>$ γ∥ $<\bar{1}11>$α′. Thus, the classical K–S was kept under both rolling conditions. The results also revealed that cryorolled deformation did not change the way of new phase generation, but affected the transition dynamics. In other words, the deformation at cryogenic temperature mainly influenced the martensitic nucleation sites and the driving force of grain growth.

### 3.3. Mechanical Properties

**Figure 5:**
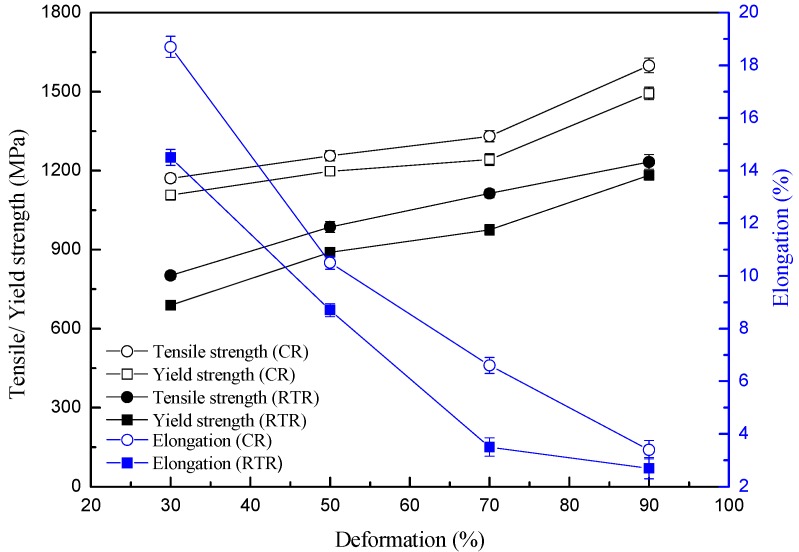
Relationship of the strength and elongation with deformations at different temperatures (the data of CR is from [14]).

Figure 5 presents the strength and elongation curves of AISI 316LN ASS rolled at room temperature and different rolling deformation. As demonstrated in [14] and in this study, both yield strength and ultimate tensile strength increased with strain under these two deformation conditions. The yield strength and ultimate tensile strength increased from 209 MPa and 527 MPa (solid solution treatment) to 1182 MPa and 1233 MPa at 90% strain. In contrast, the cryorolling led to a much higher enhancement of the elongation than its room temperature peer. After 90% cryorolling, the yield strength and ultimate tensile strength increased to 1468 MPa and 1572 MPa. This can be attributed to the suppression of the dynamic recovery of dislocations and other substructures during the cryorolling, resulting in a remarkable work-hardening effect. Furthermore, cryorolling was more favorable for the martensite transformation. As a result of martensitic transformation and work-hardening, the strength of AISI 316LN ASS after cryorolling was apparently higher at the same strain grade. This can be attributed to the emerging appearances of martensite with intrinsic high hardness subjected to cryorolling. At room temperature rolling, the hardness increased slowly following low martensite accumulation speed. Thus, the dislocation strengthening of austenite is the primary hardening mechanism. The relationship between the ultimate tensile strength and microhardness follows the empirical formula [23] where the Vickers hardness value is about 1/3 of the ultimate tensile strength value.

The trends of elongation and strain were also investigated for rolling at different temperatures. After rolling at room temperature, the elongation decreased from 83% (solid solution treatment) to 3.4% at 90% strain. The value was slightly higher than that after 90% cryorolling [14]. This was mainly because the microstructure after rolling at room temperature was a dual phase structure of austenite and martensite, while all austenite was transformed into martensite after 90% cryorolling. Below 70% strain, both austenite and martensite coexisted in the rolled ASS, with the cryorolled one having higher martensite contents. Due to the difficulty of deforming the martensite, 316LN ASS had less plasticity after cryorolling than after the room temperature treatment.

### 3.4. Fracture Morphology

**Figure 6:**
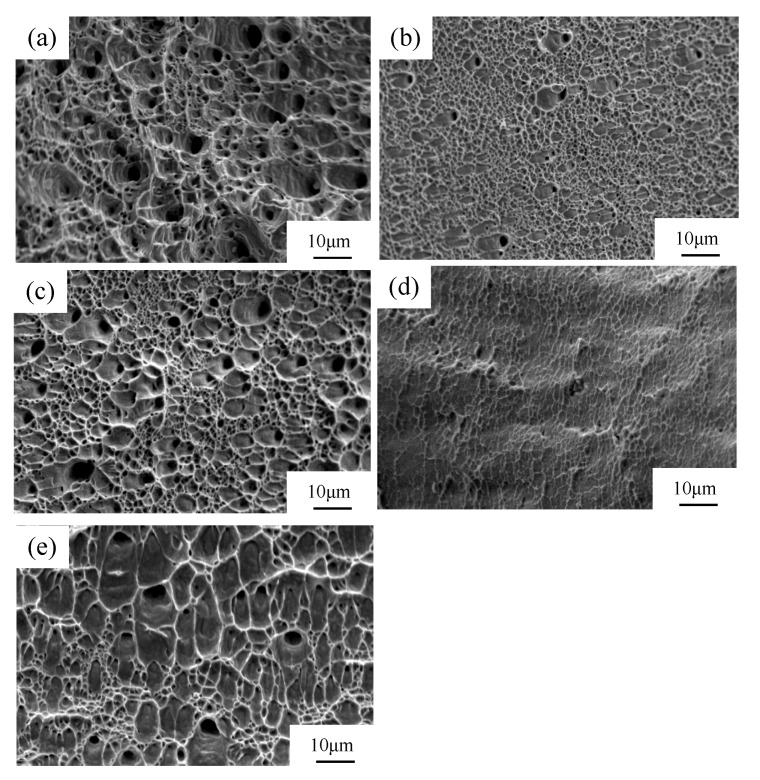
Fracture surface morphology of the 316LN austenitic stainless steel before and after rolling: (**a**) original austenite structure; (**b**) CR-30%; (**c**) RTR-30%; (**d**) CR-90%; and (**e**) RTR-90%.

The fracture morphologies after tensile tests are presented in Figure 6. Before rolling deformation, typical characteristics of ductile fracture were presented on the fracture surface of AISI 316LN ASS (Figure 6a). Though cleavage facets in different degrees were found after the rolling process, the fracture surface demonstrated a typical quasi-cleavage fracture characteristic. The occurrence of cleavage facet can be ascribed to the strain-induced martensite. In general, the proportion of cleavage facets grows constantly with the volume fraction of martensite. As indicated in Figure 6b,c, cryorolling leads to a larger proportion of cleavage facets, but with less depths and quantity of the dimples with the size of about 2 μm at the strain of 30% (CR). However, at a strain of 30% (RTR), the size of the dimples was in the range of 2~5 μm and the dimple number was higher than that at CR30. Furthermore, the fracture surface was flat and the percentage of cleavage facets decreased significantly when compared with that at CR30. At 90% strain (CR), the full martensite of the cryorolled sample led to a quasi-cleavage fracture feature where a small number of tearing ridges were distributed on the fracture surface and dimples with the size lower than 1 μm became invisible in Figure 6d. However, there existed a small number of large and deep dimples with a size of 4 μm at localized regions as well as some small and shallow dimples surrounding the cleavage facets at a strain of 90% (RTR), as shown in Figure 6e. This indicates the mixed characteristics of quasi-cleavage and ductile fracture.

## 4. Discussion

**Figure 7:**
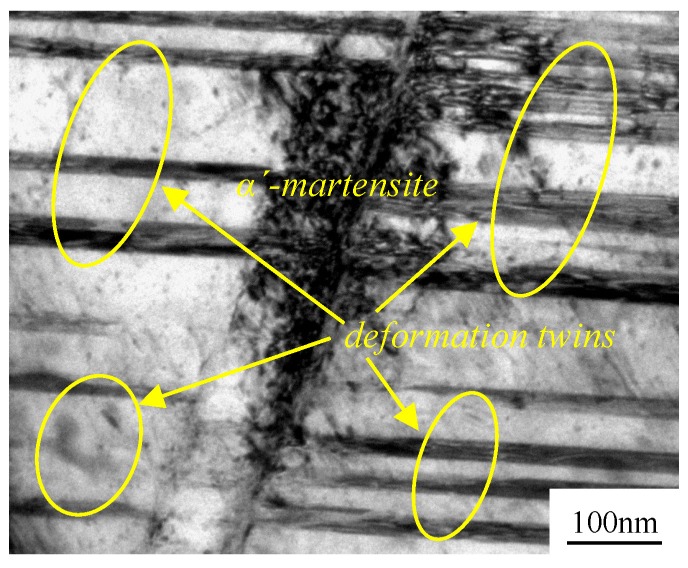
Martensite formed at the interaction position of deformation twins.

In this section, we discussed and investigated the phase transformation schemes at different rolling conditions. It was noticed that ε-martensite with a fascicle-like structure was generated due to the piling-up dislocations during the plastic deformation of the thermally meta-stable ASS. As a result, α′-martensite formed at the junctions of the fascicle-like structures [24]. However, ε-martensite was not found in the present study, and neither in the XRD patterns (Figure 1), nor in the SAED (Figure 4). Here, the primarily produced phase was α′-martensite in the AISI 316LN ASS during the rolling process, and there was little or no ε-martensite. The ε-martensite phase was also not an intermediate state during the transformation of the austenite to martensite. According to the study by Hadji [5], they verified that ε-martensite did not generate at all in severely deformed AISI 316LN ASS. Two transformation mechanisms of austenite to martensite were proposed by Sato [25] and Seetharaman [26]: (1) The transformation of martensite followed γ-austenite → ε-martensite → α′-martensite when the stacking fault energy of the base metal was below 18 mJ/m^2^; and (2) The transformation of martensite turned to γ-austenite → twin → α′-martensite when the stacking fault energy was higher than 18 mJ/m^2^. Previous research reports [27,28] stated that the stacking fault energy of AISI 316LN ASS was about 10 mJ/m^2^, indicating that the transformation of martensite in AISI 316LN ASS followed the first mechanism under the cryorolling condition. When rolling at room temperature, the sample’s temperature can rise quickly due to heat transfer in plastic deformation. Choi et al. [29] showed that the temperature of the specimen with a dual-phase ASS microstructure could be raised up to ~100 °C under uniaxial tensile testing at a strain rate of 10^−2^ s^−1^. This was also confirmed in a later study [29]. In the present study, both the strain rate and strain were much larger than those in [29,30], denoting higher temperature increases. The remarkably increased stacking fault energy could be higher than 18 mJ/m^2^ due to temperature rise [31,32]. The microstructural evolution of AISI 316LN ASS followed the second transformation mechanism during room temperature rolling, i.e., γ-austenite → twin → α′-martensite. This claim is supported by the TEM graphs in Figure 4. At the strain of 50%, a large amount of deformation twins were observed in the microstructure as shown in Figure 4d. Meanwhile, the quantity of deformation twins decreased with the strain, but the volume fraction of martensite gradually increased. Figure 7 shows the interaction of the deformation twins, which provides the nucleation site for the formation of martensite. This proved that the strain-induced martensitic transformation followed the second transformation mechanism during room temperature rolling. Cryorolling was more beneficial to martensite transformation than room temperature rolling. Besides the martensite transformation, the cryorolling also promoted the nucleation and growth of martensite. The driving force for martensite transition is the free energy difference between the newly formed and the parent phases. Deformation-induced martensite transition occurs when the sum of the deformation-induced mechanical driving force and the chemical driving force exceeds the phase transition driving force. At the same time, martensite transformation occurs through the nucleation and growth process. In this scenario, the martensite transition takes place under a supercooling temperature that is far below the martensite starting temperature. The phase transition driving force increases due to the large free energy difference, leading to the decrease of the critical nucleus size [33]. Furthermore, the dynamic recovery is significantly suppressed due to a sudden drop of atomic diffusion at cryogenic temperature. As a result, large amounts of structure defects such as dislocations and stacking faults with high energy are generated during the process of deformation. After the creation of these defects, strain-induced martensite nucleates and grows at high-density dislocation regions. Therefore, the cryorolling condition can make more martensite seeds, which further grow smoothly and deduce plenty of martensite transformed from austenite.

The large amount of quickly transformed martensite during the cryorolling process stimulated different deformation modes under the two kind of rolling conditions. Due to the rapid accumulation of strain-induced martensite under the cryorolling condition, the deformation of martensite was the dominant mechanism during the subsequent deformation process. When rolled at room temperature, the deformation of austenite dominated due to the low speed of martensite accumulation. Different microstructures were presented eventually by cryorolling and room temperature rolling at the strain of 70%. These were shown as a dual-phase microstructure containing martensite and austenite in RTR70, and as a single-phased martensite in CR70. Further increasing the strain to 90% in cryorolling, the severe plastic deformation was burdened on the martensite. It is well known that the martensite phase is very hard and difficult to deform, then during deformation, the strain-induced martensite is broken up into finer laths and presented in fiber morphology. Subjected to room temperature rolling, the softer austenite grains undertake transformations into a dual-phase microstructure, as shown by the slips along the rolling direction in Figure 3.

## 5. Conclusions

The effect of deformation temperature on the microstructure and mechanical properties of AISI316LN ASS was systematically investigated. The conclusions can be drawn as follows:

1. Strain-induced martensite transformation occurred in AISI 316LN ASS during the CR and RTR process and the volume fraction of strain-induced martensite increased with the increase of strain. At the same strain, the amount of martensite by CR was obviously higher than that by RTR. The CR was more efficient than the RTR in promoting the martensite transformation and could obtain a 100% volume fraction of α′-martensite. During both rolling processes, the orientations between the transformed martensite and the parent austenite phase followed the Kurdjumov–Sachs relationship and no orientation change was observed due to the influence of deformation temperatures.
2. The phase transition and deformation mechanism differed at the two rolling temperatures. Under the CR condition, the transformation mechanism was based on the deformation of martensite and the sequence of the transformation appeared to be γ-austenite → ε-martensite → α′-martensite. However, the main mechanism was austenite deformation during RTR and the transformation of martensite was γ-austenite → twin → α′-martensite.
3. Both CR and RTR led to a remarkable increase in the strength and hardness of the 316LN stainless steel, but to drastic reductions of the elongation. The strength and hardness after CR were higher than those after RTR, but the elongation became lower. After CR or RTR, the tensile fracture morphology changed from a typical ductile rupture (before deformation) to a mixture of quasi-cleavage and ductile fracture (after deformation).
